# SEMgsa: topology-based pathway enrichment analysis with structural equation models

**DOI:** 10.1186/s12859-022-04884-8

**Published:** 2022-08-17

**Authors:** Mario Grassi, Barbara Tarantino

**Affiliations:** Department of Brain and Behavioral Sciences, University of Pavia, Pavia, Italy

**Keywords:** Pathway enrichment analysis, Pathway topology, SEM, SEMgsa, Sensitivity, Prioritization, Type I error, Power

## Abstract

**Background:**

Pathway enrichment analysis is extensively used in high-throughput experimental studies to gain insight into the functional roles of pre-defined subsets of genes, proteins and metabolites. Methods that leverages information on the topology of the underlying pathways outperform simpler methods that only consider pathway membership, leading to improved performance. Among all the proposed software tools, there’s the need to combine high statistical power together with a user-friendly framework, making it difficult to choose the best method for a particular experimental environment.

**Results:**

We propose SEMgsa, a topology-based algorithm developed into the framework of structural equation models. SEMgsa combine the SEM *p* values regarding node-specific group effect estimates in terms of activation or inhibition, after statistically controlling biological relations among genes within pathways. We used SEMgsa to identify biologically relevant results in a Coronavirus disease (COVID-19) RNA-seq dataset (GEO accession: GSE172114) together with a frontotemporal dementia (FTD) DNA methylation dataset (GEO accession: GSE53740) and compared its performance with some existing methods. SEMgsa is highly sensitive to the pathways designed for the specific disease, showing low *p* values ($< 0.001$) and ranking in high positions, outperforming existing software tools. Three pathway dysregulation mechanisms were used to generate simulated expression data and evaluate the performance of methods in terms of type I error followed by their statistical power. Simulation results confirm best overall performance of SEMgsa.

**Conclusions:**

SEMgsa is a novel yet powerful method for identifying enrichment with regard to gene expression data. It takes into account topological information and exploits pathway perturbation statistics to reveal biological information. SEMgsa is implemented in the R package SEMgraph, easily available at https://CRAN.R-project.org/package=SEMgraph.

**Supplementary Information:**

The online version contains supplementary material available at 10.1186/s12859-022-04884-8.

## Background

Biomedical research has been transformed by recent advances in high-throughput technologies, enabling extensive monitoring of complex biological systems. As a result, new methodological developments have emerged, most notably the adaptation of systems perspectives to analyze biological systems. Pathway enrichment has become a key tool in the analytic pipeline for Omics data and has been effectively used to generate novel biological hypotheses and determine if specific pathways are linked to specific phenotypes. In the literature, dozens of strategies have been developed, varying in model complexity and effectiveness.

Earlier methodologies, such as over-representation analysis (ORA) [1] and gene set analysis (GSA) [2, 3], treat each pathway as a collection of biomolecules, as [4] point out in their review paper. The ORA approach used a list of differentially expressed (DE) genes as input to determine which sets of DE genes are over-represented or under-represented, being strongly reliant on the criteria used to choose the DE genes, such as the statistical tests and thresholds utilized.

A second generation of approaches was created to reduce this reliance on gene selection criteria by taking into account all gene expression values. The hypothesis behind these approaches is that small yet coordinated changes in groups of functionally related genes may be crucial in biological processes. These methods are named functional class scoring methodologies (FCS) [5]. Such methods include gene set enrichment analysis (GSEA) [2], gene set analysis (GSA) [6] and correlation adjusted mean rank gene set test (CAMERA) [7] among others.

ORA and FCS methods can be referred to as the first two generations of pathway enrichment analysis approaches. However, when pathways are seen as a basic unstructured and unordered collection of genes, all the genetic connections and interactions that are supposed to capture and characterize the actual processes at hand are simply neglected.

With the aim of including all of this additional information into the analysis, topology-based (TB) approaches have been created. These methods account for interactions between biomolecules and provide better performance than standard second-generation methods [2, 3]. A variety of tools have been implemented, such as DEGraph [6], topologyGSA [8], NetGSA [9–11], Pathway-Express [12, 13], SPIA [14] among others. The common feature of approaches in this category is that they use prior knowledge of pathway topology information to obtain some gene-level statistic, which is then used to produce a pathway-level statistic, which is then used to rank the pathways.

The goal of pathway enrichment approaches is to compare the ’activity’ of interest pathways across two or more biological situations or groups of specimens (patients, cell lines, etc.). Another technical feature that distinguished pathway enrichment methods is the type of the statistical null hypothesis being tested. The majority of approaches may be divided into two categories: those that test (I) self-contained null hypotheses and (II) competitive null hypotheses [15]. A self-contained null hypothesis examines the activity of each pathway across biological situations (for example, normal vs. illness samples) without comparing it to the activity of other biomolecules/pathways. On the other hand, the activity of each pathway is compared to that of other biomolecules/pathways in a competitive null hypothesis. Even if the competitive null hypothesis has an interesting interpretation, assessing the significance of the competitive null is challenging, since tests based on it take into account a framework for gene sampling that treats genes as independent.

The main contribution of this article is the development of a self-contained topology-based algorithm developed into the framework of structural equation models (SEM), called SEMgsa() [16, 17]. Evaluation of system perturbation is incorporated in SEM [18], where the experimental condition is compared to a control one through the use of an exogeneous group variable acting on every node of the network. Statistical significance of specific-pathway score is obtained combining node activation and node inhibition statistics extracted from SEM model fitting. In addition, unlike existing methods, an overall status of pathway perturbation of genes between case and control group has been computed considering both node perturbation and up- or down- regulation of genes for gaining more biological insights into the functional roles of predetermined gene subsets.

A second objective of this study is to provide a consistent optimum solution of any given biological situation. Many topology-based methods that investigate distinct null hypothesis have been proposed in literature. We compare five popular pathway analysis approaches to SEMgsa(), starting from the most similar ones in terms of multivariate test and self-contained hypothesis type (DEGraph, NetGSA and topologyGSA) together with one approach of competitive hypothesis type (PathwayExpress) and the older approach of over-representation analysis (ORA). All the methods in this article offer a nice use interface in R.

The aforementioned methods have been applied on observed and simulated expression data. The ultimate goal of expression data application is to provide a meaningful comparison of gene set analysis methods in terms of (i) sensitivity and (ii) prioritization for observed data and (i) type I error and (ii) power within each simulation run.

The remainder of the article is organized as follows. Firstly, we describe SEMgsa() features with regard to gene expression data both in terms of inference procedure and user interface. Then, we outline the experimental setup constructed to evaluate pathway enrichment methods, including real data application and simulation design. In the end, we provide the results together with overall discussion.

## Method and implementation

### SEM framework

A structural equation model (SEM) [18, 19] is a statistical framework for causal inference based on a system of structural equations defining a *path diagram*, represented as a graph $G = (V, E)$, where *V* is the set of nodes (i.e., variables) and *E* is the set of edges (i.e., connections). The set *E* may include both directed edges $k \rightarrow j \, \, {\mathrm {if}} \, \, k \in {\mathrm {pa}}(j)$ and bidirected edges $k \leftrightarrow j \, \, {\mathrm {if}} \, \, k \in {\mathrm {sib}}(j)$. Although in the general setting of SEM latent variables and non-linear functions can be included [18], we focus on the special case where the *parent* set ${\mathrm {pa}}(j)$, and the *siblings* set ${\mathrm {sib}}(j)$, determine a system of *linear* equations, as follows:

1 $$\begin{aligned} Y_j&= \displaystyle \sum _{k \, \in \, {\mathrm {pa}}(j)} \beta _{jk} Y_k + U_j \qquad j \in V \end{aligned}$$

2 $$\begin{aligned} {\mathrm {cov}}(U_j; U_k)&= {\left\{ \begin{array}{ll} \psi _{jk} & \quad {\mathrm {if}} \, j = k \, \, \,{\mathrm {or}} \, \, k \in {\mathrm {sib}}(j)\\ 0 & \quad \text {otherwise} \end{array}\right. } \end{aligned}$$

where $Y_j$ and $U_j$ are an observed variable and an unobserved error term, respectively; $\beta _{jk}$ is a regression (path) coefficient, and a covariance $\psi _{jk}$ indicates that errors are dependent, which is assumed when there exists an unobserved (i.e. latent) confounder between *k* and *j*.

Under such a model, the dependence structure among genes provided by pathways in biological database with directed and/or bi-directed edges (i.e., KEGG, Reactome, and many others) [20, 21], interacting with each other to generate a single biological effect, can be included explicitly though the graph, $G=(V,E)$ and evaluated using local and global statistics.

**Fig. 1:**
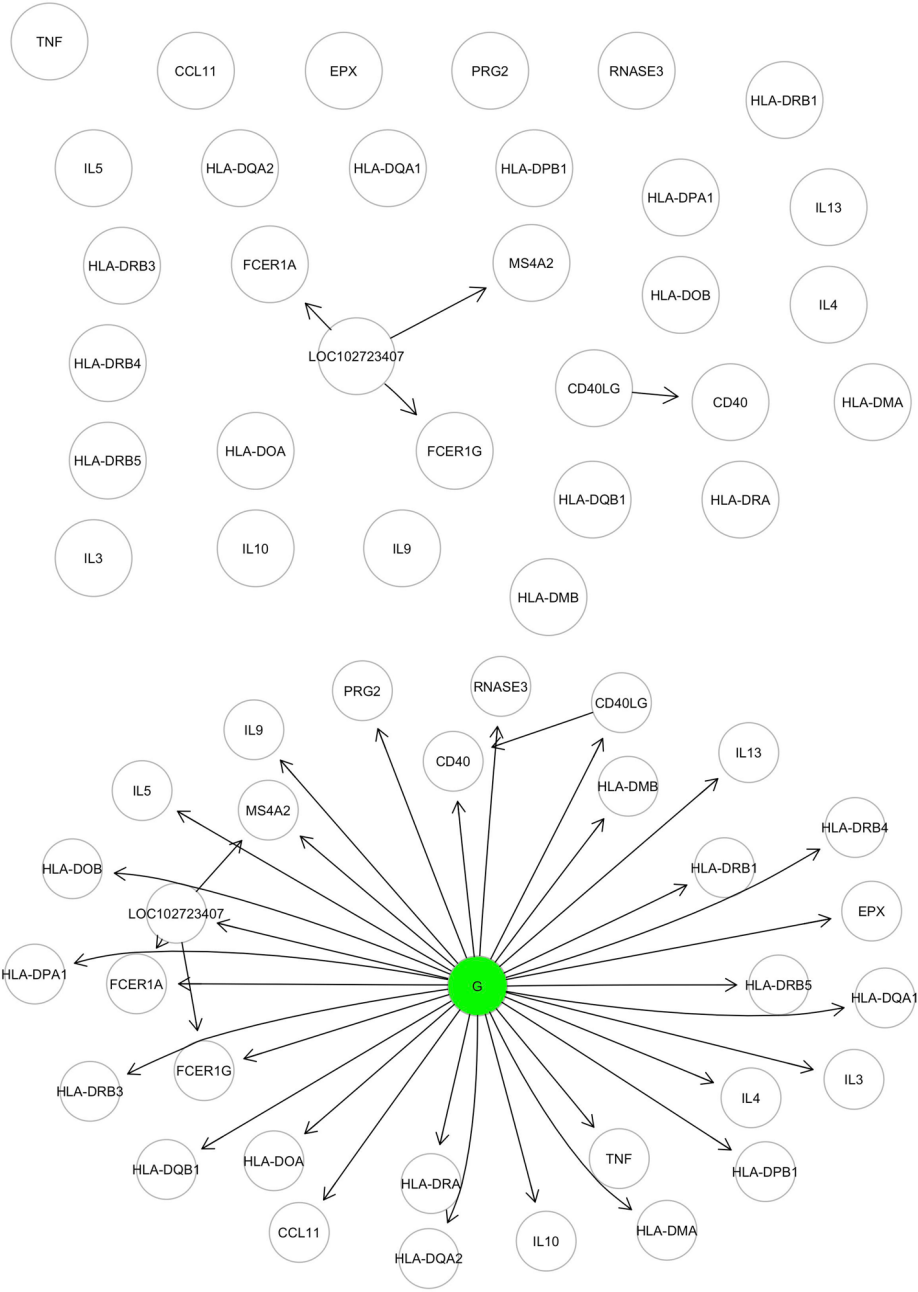
Visualisation of SEMgsa() procedure starting from Asthma KEGG pathway. The first graph summarise Asthma network properties, showing a pathway consisting of 31 nodes, 4 edges and 25 singletons. To maximise pathway information, SEMgsa procedure adds a binary group node (G = {0, 1}) that directly affects the set of genes in the pathway. In this way, the pathway with numerous singleton genes is edged with a group node and group-genes, resulting in a linked graph

SEMgsa() procedure adds a binary group (treatment or disease class) node labeled *X* to *V*, and suppose that $X = \{0, 1\}$ directly affects the set of genes in the pathway. As bonus, adding group node and group-genes edges, the pathway with several components (clusters) and singleton genes induces a connected graph (see Fig. 1). Thus we consider a directed graph $G=(V \cup {X}, E \cup E_{X})$ with the linear structural equations:

3 $$\begin{aligned} Y_j&= \beta _{j} X + U_j \qquad j \in V(x) \end{aligned}$$

4 $$\begin{aligned} Y_j&= \displaystyle \sum _{k \, \in \, {\mathrm {pa}}(j)} \beta _{jk} Y_k + \beta _jX + U_j \qquad j \in V(y) \end{aligned}$$

where *V*(*x*) and *V*(*y*) are the sets of exogenous (i.e., source and singleton genes) and endogenous (i.e., connectors and sinks) genes, respectively. The covariances, ${\mathrm {cov}}(U_{j}; U_{k})$ are assumed to be equal to Eq. (2).

Comparing Eq. (1) with Eqs. (3)–(4), we note that the added node *X* may affect the mean gene expression values, but not their regression paths or covariances. In the R package **SEMgraph** these coefficients can vary by experimental or disease group via two-group SEM [16, 17], but in the following we assume additive group effects. Coefficients $\beta _j$ (adjusted by the parents of the *j*-th node) determine the effect of the group on the *j*-th node, while the common path coefficients $\beta _{jk}$ represent regression coefficients, adjusted by parent set and group effect.

This type of SEM enables the identification of differentially expressed genes (DEGs) if genes show a statistically significant variation in their activity (e.g., gene expression) in the experimental group respect to the control one. In other terms, a test for the null value of the path, $X \rightarrow Y$ is a test of:

5 $$\begin{aligned}&H_0:Y_j \perp \left\{ X \right\} \,\,\, vs. \,\,\, H_1:Y_j \not \perp \left\{ X \right\} , \,\,\, j \in V(x) \end{aligned}$$

6 $$\begin{aligned}&H_0:Y_j \perp \left\{ {\mathrm {pa}}({\text{Y}}_{\text{j}}),X \right\} \,\,\, vs. \,\,\ H_1: Y_j \not \perp \left\{ {\mathrm {pa}}({\text{Y}}_{\text{j}}),X \right\} , \,\,\, j \in V(y) \end{aligned}$$

From Eqs. (5)–(6) we note two different tests. Marginal tests of conventional DEGs analysis [22] for source and singleton genes, and conditional tests, given the parents, for connectors and sink genes. Conditioning increases power when there is a direct group effect, and reduces gene variability. So, pathway topological structure makes the inference more precise [23].

Maximum likelihood estimates (MLEs) of the paths ($X \rightarrow Y$) can be easily obtained with one of the three algorithms of **SEMgraph**. Specifically, the core of model fitting in SEMgsa() function relies on the residual iterative conditional fitting (RICF), an efficient iterative algorithm that can be implemented through least squares, with the advantage of clear convergence properties [24], and permutation-based *p* values for testing null hypothesis in Eqs. (5)–(6).

*p* values of group effect ($X \rightarrow Y$) are computed by randomization of group labels comparing the estimated parameters by RICF with their random resampling distribution after a sufficiently high number of case/control labels permutations. Accurate small *p* value estimations are possible with no need for a large number of permutations (SEMgsa() makes default = 1000 permutations), using the moment based approximation proposed by [25]. Once the empirical distribution of the permuted path coefficients is obtained, the two-sided *p* values are extracted from the normal distribution with mean and standard deviation estimated from the empirical distribution.

From node-wise *p* values, overall group perturbation on pathway genes can be computed based on the Brown’s method for combining non-independent, one-sided significance tests [26]. The method computes the sum of one-sided *p* values: $X^2 = -2 \sum _{j} {\mathrm {log}}(p_j)$, where the direction is chosen according to the alternative hypothesis (${\mathrm {H}}_1$), and the overall *p* value is obtained from the chi-square distribution with new degrees of freedom *f* and a correction factor *c* to take into consideration the correlation among *p* values, resulting in $\frac{X^2}{c}\sim {\chi }^2(f)$.

The conversion of two-sided *p* values in one-sided *p* values is performed according to the sign of the path ($X \rightarrow Y$) coefficient, $\beta _j$:

7 $$\begin{aligned}&{\mathrm {H}}_1{:}\, {\text {with}} \,{\text {at}}\,{\text { least}}\, {\text {one}} \, \, \beta _j> 0 \implies p_j^{(+)} = {\left\{ \begin{array}{ll} p_j / 2 & \quad {\mathrm {if}} \, \beta _j > 0\\ 1 - p_j / 2 & \quad {\mathrm {if}} \, \beta _j < 0 \end{array}\right. } \end{aligned}$$

8 $$\begin{aligned}&{\mathrm {H}}_1{:}\, {\text {with}}\, {\text {at}}\, {\text {least}}\, {\text {one}} \, \, \beta _j< 0 \implies p_j^{(-)} = {\left\{ \begin{array}{ll} p_j / 2 & \quad {\mathrm {if}} \, \beta _j < 0\\ 1 - p_j / 2 & \quad {\mathrm {if}} \, \beta _j > 0 \end{array}\right. } \end{aligned}$$

If the overall *p* value $< \alpha$ (i.e., the significance level), we define node perturbation as *activated* when the direction of the alternative hypothesis is positive. Conversely, the status is *inhibited* if the direction is negative.

Node-wise *p* values $< \alpha$ (after correcting for multiple comparisons with one of several adjustment methods, including Bonferroni or Benjamini–Hochberg procedure), are used for DEGs identification. While, a single *p* value of the two Brown’s *p* values ($p^{(+)}$: p-activation, and $p^{(-)}$: p-inhibition) combined with a Bonferroni procedure [27], i.e. $2\times min(p^{(+)}; p^{(-)})$, indicates the global pathway perturbation.

In some cases, edge weights are defined in signalling pathways with discrete values [$-1, 0, 1$], indicating gene-gene activity derived from biological database (e.g. KEGG). Usually they are: $-1$ for repressed or inactive, 0 for neutral, and + 1 for enhanced or activated. For gaining more biological insights into the functional roles of prior subset of genes, the sign of the minimum *p* value between node activation and inhibition has been retained to assess, in combination with pathway weights, an overall status of pathway perturbation of genes between case and control group. In detail, node perturbation obtained from RICF fitting has been combined with up- or down-regulation of genes to obtain overall pathway perturbation classification as displayed in Table 1.

**Table 1:** Overall pathway perturbation

Up/down regulation	Node perturbation	Overall perturbation
+ 1	P− (inh)	Down act
− 1	P− (inh)	Up inh
+ 1	P+ (act)	Up act
− 1	P+ (act)	Down inh

- The weighted adjacency matrix of each pathway was used to determine the up- or down-regulation of genes (taken from the KEGG database) as the column sum of weights across each source node. The pathway is marked as down-regulated if the total sum of the node weights is less than 1, and otherwise as up-regulated.
- The minimum among the *p* values determines whether the node perturbation is activated or inhibited; if positive, the node perturbation is described as activated, and otherwise as inhibited.
- It is possible to determine the direction (up or down) of gene perturbation by combining these two quantities. In cases compared to the control group, an up- or down-regulated gene status that is associated with node inhibition shows a decrease in activation (or an increase in inhibition). In contrast, up- or down-regulated gene status, associated with node activation, leads to an increase in activation (or decrease in inhibition) in cases relative to control group.

### User interface

The example code of the function SEMgsa() is as follows.

SEMgsa(g = list(), data, group, method = "BH", alpha = 0.05, n_rep = 1000, ...)

The inputs are: a list of pathways to be examined (*g*); gene expression data where rows represent subjects, and columns graph nodes (*data*); a binary vector with 1 for cases and 0 for control subjects (*group*). Optional inputs are the multiple testing correction method (*method*), and the significance level (*alpha*) for DEGs selection, and the number of randomization replicates for RICF algorithm ($n\_rep$, default = 1000).

The first step in SEMgsa() workflow is to compute the weighted adjacency matrix of each pathway, obtain the sum of node weights and flag the pathway as up- or down-regulated. This is crucial to obtain the overall pathway perturbation status at the end. Then RICF algorithm of R package **SEMgraph**, i.e. SEMrun(graph, data, group, fit = 1, algo = "ricf"), is applied on data, considering the group binary vector and the number of specified randomization replicates. More specifically, SEMrun() takes as input a single *graph* as an *igraph* object and has several additional inputs, including: a numeric value *fit* indicating the SEM fitting mode, where $fit = 1$ specifies a “common” model to evaluate group effects on graph nodes; the MLE method *algo* is used for model fitting, in this case fitting is done via *RICF*(*algo* = “*ricf*”).

The covariance matrix could not be semi-definite positive in the situation of large dimensionality (n.variables > n.subjects), making it impossible to estimate the parameters. When this occurs, regularization of the covariance matrix is enabled. SEMrun() uses internally two functions of the corpcor R package: the is.positive.definite() tests if the observed covariance matrix is positive, and if the response is equal to FALSE, the function pcor.shrink() implements the James-Stein-type shrinkage estimator [28] to regularized the covariance matrix.

Node-wise group effect *p* values are extracted from model fitting object together with the number of DEGs obtained adjusting *p* values with the chosen correction *method* while testing the specified level of *alpha*. Then, a data frame of combined SEM results is obtained putting together node-wise *p* values with Brown’s method and Bonferroni’s correction.

The output of SEMgsa() is represented by a list containing two objects with the following information for each pathway in the input list:

- **gsa**, a dataframe reporting size (*No*.*nodes*), DEGs number (*No*.*DEGs*), pathway perturbation status (*pert*), Brown’s combined *p* value of pathway node activation (*pNA*), Brown’s combined *p* value of pathway node inhibition (*pNI*) and the Bonferroni combination of them (*PVAL*). *ADJP* refer to the pathway combined *p* value adjusted for multiple tests with Bonferroni correction, i.e., $ADJP = min(K \times PVAL; 1)$, where *K* is the number of the input pathways.
- **DEG**, a list with DEGs names for each pathway, selected with *p* value < *alpha* after the multiple correction procedure with one of the method available in R function p.adjust(). By default, *method* is set to “BH” (i.e., Benjamini–Hochberg correction) and the significance level *alpha* to 0.05.

To read more about SEMgsa() function, in terms of description, usage, function arguments and value, refer to https://rdrr.io/cran/SEMgraph/man/SEMgsa.

## Experimental design

### Benchmark data

Coronavirus disease (COVID-19) RNA-seq expression data from [29] (GEO accession: GSE172114) together with Frontotemporal Dementia (FTD) DNA methylation data (DNAme) from [30] (GEO accession: GSE53740) have been used as benchmark data. Network information has been retrieved from kegg.pathways object of the **SEMgraph** package as a list of 225 edge weighted *igraph* objects corresponding to the KEGG pathways extracted using the *ROntoTools* R package [31]. Edge weights are defined with discrete values $[-1, 0, 1]: -1$ for inactive gene–gene activity, 0 for neutral, and $+1$ for activated.

#### Coronavirus disease (COVID-19)

Coronavirus disease of 2019 (COVID-19) is a highly contagious respiratory infection that is caused by the severe acute respiratory syndrome coronavirus 2 (SARS-CoV-2). Multiple probes for each Entrez gene ID were first eliminated. The empirical Bayes technique, as implemented in the *limma* R package [32], was used to fit linear models for differential expression analysis, and *p* values were adjusted for multiple testing using the method of Benjamini–Hochberg [33]. This procedure results in a matrix of 69 subjects $\times$ 14,000 genes. Subjects include patients in the intensive care unit with Acute Respiratory Distress Syndrome (“critical group”, N = 46) defined as cases, and those in a non-critical care ward under supplemental oxygen (“non-critical group”, N = 23) defined as controls. The expression matrix was finally matched with the corresponding Coronavirus disease—COVID-19 (*hsa*05171) KEGG pathway according to its name. The latter is a graph with 232 nodes and 208 edges, including five components and 109 sigleton (i.e. node degree = 0). The maximum subgraph consists of 54 nodes and 83 edges. This pathway was subsequently labeled as target pathway and its *p* value and rank were further investigated for assessing the sensitivity and prioritization ability of the methods [34, 35].

#### Frontotemporal dementia (FTD)

Frontotemporal Dementia, a neurodegenerative disorder characterized by cognitive and behavioural impairments [36]. We will use DNAme data stored in the **SEMdata** package as ftdDNAme, a list of two objects: a data matrix of 256 rows (subjects) and 16,560 columns (genes) containing the value of the first principal component of DNAme levels, obtained applying a principal component analysis to methylated CpG sites within the promoter region, for each gene (genes with unmethylated CpGs in both conditions were discarded); and a binary group vector of 105 FTD patients (1) and 150 healthy controls (0). Unlike COVID-19 data, FTD has not a unique KEGG pathway associated to its name. According to KEGG BRITE database, the term Frontotemporal lobar degeneration (an alias for FTD; KEGG ID:H00078) is associated to 6 KEGG pathways: MAPK signaling pathway (*hsa*04010), Protein processing in endoplasmic reticulum (*hsa*04141), Endocytosis (*hsa*4144), Wnt signaling pathway (*hsa*04310), Notch signaling pathway (*hsa*04330), and Neurotrophin signaling pathway (*hsa*04722). We can use the SEMgsa() function to apply gene set analysis (GSA) on a collection of networks, exploring the 6 selected FTD pathways as target ones. Thus, the ability of GSA methods will be investigated on 6 target pathways, combining results in terms of median *p* values and ranks for readability purposes. We refer the reader to the Additional file 1 for more detailed results.

### Data simulations

Synthetic data, based on realistic expression data (“Coronavirus disease (COVID-19)” section), was used to carry out simulations following the practice in [10]. A subset of pathways $q_{1} < K$ out of total *K* pathways has been chosen to be dis-regulated. Next, a pre-specified number (*s*) of genes within each dis-regulated pathway was chosen to be altered (up or down) according to a topological measure (betweenness, community, neighbourhood) and different mean signals ($\pm 0.5, \pm 0.6, \pm 0.7$). Another subset of $q_{0} < (K - q_{1})$ pathways with no dis-regulated genes has been chosen to evaluate statistical metrics. Finally, the COVID-19 benchmark data matrix is first normalized, with mean zero and unit variance for each gene within each group (cases and controls). Then, nine data generation procedures are executed, according to topological measures and adding mean signal to the pre-specified genes in the selected dis-regulated pathways. In summary, the simulation design ($3 \times 3$) with 100 randomization per design levels is reported in Table 2.

**Table 2:** Summary of simulation design ($3 \times 3$) with 100 randomization per design levels

Topology design	Gene regulation	Mean signal		
		$\pm$ 0.5	$\pm$ 0.6	$\pm$ 0.7
Betweenness	Up/down	100	100	100
Community	Up/down	100	100	100
Neighbourhood	Up/down	100	100	100

#### Pathway deregulation

Each data generation procedure starts with the definition of a list of pathways to be tested. After recalling the list of kegg.pathways including $N=225$ signaling pathways from the KEGG database, for efficiency purposes pathways with a minimum and a maximum number of nodes equal respectively to 30 and 300 have been filtered out for the analysis. Then, to speed up computations, the maximum component of each *igraph* [37] object corresponding to each selected pathway has been selected. Given this choice, *igraph* objects with maximum component smaller than the $60\%$ of the total graph size have not been considered. This filtering procedure results into a list of $K=117$ *igraph* objects.

We have to alter $q_{1}=10$ pathway’s genes in order to deregulate it within a simulation. Specifically, we consider the 9 KEGG pathways associated to Coronavirus disease—COVID-19 (*hsa*05171): Vascular smooth muscle contraction (*hsa*04270), Platelet activation (*hsa*04611), Toll-like receptor signaling pathway (*hsa*04620), NOD-like receptor signaling pathway (*hsa*04621), RIG-I-like receptor signaling pathway (*hsa*04622), JAK-STAT signaling pathway (*hsa*04630), Natural killer cell mediated cytotoxicity (*hsa*04650), Fc gamma R-mediated phagocytosis (*hsa*04666) and Leukocyte transendothelial migration (*hsa*04670).

We looked at three different methods for reflecting pathway topology in order to assign impacted genes to the deregulated pathways: betweenness, community and neighborhood, following the practice in [10, 38].

The number of all shortest paths in a directed graph that pass through a given node is known as its betweenness. The top 10 highest scoring betweenness nodes were used to choose affected genes in the *betweenness deregulation* design. According to the *community deregulation* design, we located modules with dense connections between module nodes and spare connections between module nodes. Given the division of vertices in each community, the 10 affected genes are then randomly sampled from the community with the highest proportion of members. In the *neighbourhood deregulation* procedure, the vertices not farther than a given limit from another fixed vertex are called the neighborhood of the vertex. After computing the neighbourhood of order 2, we sampled the 10 vertices from the neighbourhood with the biggest size.

Within each of the associated pathways plus the target pathway of interest, an equal number of genes *s* has been selected as ‘dysregulated’. We decided to fix the number of affected genes to $s=10$ for each pathway, with the aim of obtaining equal contribution from each associated disease (due to the presence of smaller pathway sizes). However, given that overlapping genes between pathways may occur, the unique genes out of the total $S \le s \times q_{1} =10 \times 10$ have been retained as DEGs for pathway dysregulation. Thus, the number of total dysregulated genes *S* is not fixed, but changes according to the chosen topology design. As a note, given the random sampling within the community deregulation design, the sampled genes change according to the specified seed. In the end, a weight representing the up- or down-regulation of genes was kept along with the Entrez gene ID of the affected genes. The weighted adjacency matrix of each pathway comprises a column regarding the sum of weights over each source node, which can be used to determine up- or down-regulation (taken from the KEGG database). The pathway is marked as down-regulated or up-regulated depending on whether the total sum of node weights is less than 1. Each gene’s weight has been extracted in order to obtain an up or down mean signal, which better reflects variations in the expression of the impacted genes between the control and treatment groups.

After identifying the subset of DEGs according to the chosen topology design, pathways with a number of dysregulated genes $\le 1$ have been selected as true negatives ($q_{0}$) to evaluate type I error from simulations. Unlike the $q_{1}$ number that is fixed to 10 COVID-19 related pathways, the number of $q_{0}$ pathways changes according to the chosen subset of DEGs.

To summarise, for all topology design, the total altered genes *S* inside the $q_1=10$ pathways are differentially expressed with a mean difference varying from ($\pm 0.5$, $\pm 0.6$, $\pm 0.7$). Note that the magnitude of the mean signal is expressed relative to the unit variance of each gene (see [10]).

The simulated expression matrices were directly supplied to SEMgsa() and DEGraph, topologyGSA, NetGSA, PathwayExpress and ORA algorithms together with the list of *igraph* objects corresponding to the chosen KEGG pathways.

#### Pathway enrichment methods

Table 3 provides an overview of the tested pathway enrichment methods in terms of null hypothesis, input requirements, pathway information and availability on R together with main papers for reference. These methods differ in two main aspects: (i) the type of null hypothesis, self-contained or competitive; (ii) input data, expression data or thresholded gene *p* values. ORA and PathwayExpress test the competitive null hypothesis of whether the genes in the set of interest are at most as often DE as the genes not in the set, instead SEMgsa(), DEGraph, topologyGSA and NetGSA test the self-contained null hypothesis of no genes in the set of interest are DE. Another major difference among these methods regards the input requirements. There is a high sensitivity to the *p* value cutoff because all techniques based on testing the competitive null hypothesis must identify DE genes based on a pre-specified threshold of corrected *p* values. Without making any arbitrary decisions on the list of DE genes, all self-contained tests directly employ expression data.

For computational purposes, two main aspects have been addressed within the main analysis, mostly regarding DEGraph, topologyGSA and NetGSA:

- *Common covariance matrix*: DEGraph, TopologyGSA and NetGSA are multivariate hypothesis testing-based approaches. The vectors of gene expression levels in each (sub)pathway are assumed to be random vectors with multivariate normal distributions, $N_{p}(\mu _{1},\Sigma _{1})$ and $N_{p}(\mu _{0},\Sigma _{0})$ where the covariance matrix stores the network topology information. If the two distributions of the gene expression vectors corresponding to the two phenotypes differ significantly from one another, the network is thought to be strongly altered when comparing the two phenotypes. A multivariate hypothesis test is used to determine significance. The key distinctions between these three analytic approaches are the specification of the null hypothesis for statistical tests and the procedures for calculating the parameters of distributions.
  DEGraph was developed to perform a two-sample test of means while taking the topology of the genes into account. It considers a special case where both covariance matrices are expected to be equal, $\Sigma _{1}$ = $\Sigma _{0}$ and tests the null $\mu _{1}$ = $\mu _{0}$. DEGraph uses a modified multivariate Hotelling $T^{2}$-test hypothesis to identify significant (sub)pathways. Two groups are compared in terms of the first k components of the graph-Fourier basis (or in the original space after filtering out k high-frequency components). In our analysis, the largest component is used as a representation of the whole pathway.
  TopologyGSA begins by transforming the pathway network graph into a direted acyclic graph (DAG) and then to its “moral” graph by connecting all parent nodes of a vertex and removing the edge directionality. The moral graph is then decomposed into cliques (i.e. subsets of nodes in the graph for which each pair is connected by an edge). A set of two hypothesis tests is applied to compute the statistical significance of the impact on a given graph. The first test determines if the inverses of the covariance matrices are equal. The second hypothesis test examines the equality of the distributions’ means. To reduce the computational burden of this method, we consider a special case like DEGraph by assuming that the covariance matrices are expected to be equal and we employed the hypothesis test only for the mean of the distributions. When the covariance matrices are equal, the test of differential expression for the means is performed through a multivariate analysis of variance (MANOVA), equivalent to Hotelling’s $T^{2}$-test.
  With NetGSA, the *K* networks may differ, and *K* take into account a linear mixed effects model for each condition. The underlying biological network is encoded in the 0–1 adjacency matrix, $A_{k}$ which determines the influence matrix $\Lambda _{k}$ under each condition. The latter matrix describes the impact of each gene on all the other genes in the network and is calculated from the adjacency matrix, $\Lambda _{k} = (I - A_{k})^{-1}$. Here we defined $k = 2$ and, to speed up computations, we assumed that the network is shared between the two conditions, allowing the computation of only one common adjacency matrix for case and controls.
- *Hotelling’s* $T^{2}$*-test*: this test represent a a natural generalisation of the t-test for testing the difference between multivariate means of two populations. $T^{2}$ is equivalent to Mahalanobis distance: $D^{2} = ({\bar{y}}_{1} - {\bar{y}}_{0})^{T} {\hat{\Sigma }}^{-1}({\bar{y}}_{1} - {\bar{y}}_{0})$, where ${\hat{\Sigma }}$ is an estimation of the common covariance matrix. It is known to be consistently most effective against global mean-shift alternatives for multivariate normal distributions, but it may exhibit poor behavior in high dimensions. The $T^{2}$ test has very poor performance when the number of genes, p is close to number of samples, n; and is ill-defined when p equals or exceeds n. This is because the test statistic relies on the inverse of the estimated covariance matrix, which does not exist when p $\ge$ n and has large variation when p is close to n . We proposed to regularize the sample covariance matrix in order to to stabilize its inverse and we used the decorrelated mean difference, as test statistic: $D = ({\bar{y}}_{1} - {\bar{y}}_{0})^{T} {\hat{\Sigma }}^{-\frac{1}{2}} u/\sqrt{p}$, where $u = (1, 1, \dots , 1)$. *D* are very close to $D^{2}$ [5], but the former is computationally more efficient, especially if randomization procedure of the null distribution is used. In this way, we solved issues regarding the computation of $T^{2}$ test for both topologyGSA and DEGraph. In addition, permutation-based *p* values for testing null hypothesis are computed by randomization of group labels, as performed in SEMgsa() function, allowing more accurate *p* value estimation with no need of a large number of permutations.

**Table 3:** Overview of tested pathway enrichment methods

Method	Null hypothesis	Gene *p* value tresholding	Expression data	Pathway	R/Bioconductor [References]
SEMgsa	Self-contained	No	Yes	Topology	SEMgraph 1.1.1 [17]
DEGraph	Self-contained	No	Yes	Topology	DEGraph 1.46.0 [14]
TopologyGSA	Self-contained	No	Yes	Topology	TopologyGSA 1.4.7 [13]
NetGSA	Self-contained	No	Yes	Topology	netgsa 4.0.3 [10–12]
PathwayExpress	Competitive	Optional	No	Topology	ROntoTools 2.23.0 [6, 8]
ORA	Competitive	Yes	No	Membership	EnrichmentBrowser 2.25.3 [1]

#### Evaluation measures

In the benchmark data analysis, all methods were evaluated according to (i) sensitivity and (ii) prioritization. The sensitivity refers to the ability of producing small *p* values for the target pathway and prioritization refers to the ability of ranking close to the top the gene sets that are indeed relevant to a given condition.

Performance of GSA methods within each simulation run has been evaluated looking at (i) type I error and (ii) power. When a true null hypothesis is rejected, a type I error, also known as a false positive, occurs, whereas the power assesses the probability of a test successfully rejecting the null when the alternative hypothesis is true. Power comparisons are only useful if the tests have appropriate type I error control. Pathways associated with COVID-19 disease ($q_{1}$) were used to evaluate power, whereas those with a number of dysregulated genes $\le 1$ ($q_{0}$) were utilized to evaluate type-I error [11].

The type I errors and powers were estimated as the fraction of null hypotheses rejected across 100 simulated replications.

## Results

### Benchmark results

Significance of target pathway was detected if the adjusted *p* value (after Bonferroni correction for multiple tests) didn’t exceed the level of 0.05. COVID-19 pathway was identified only by SEMgsa() together with topologyGSA and NetGSA. The lowest *p* value was reported by SEMgsa() together with topologyGSA (see Table 4). DEGraph, ORA and PathwayExpress seems not to be sensitive to mean changes between conditions in Coronavirus data. Looking at the results for Frontotemporal Dementia, the only significant *p* value (in terms of median *p* values of related pathways) is reported by SEMgsa(), confirming the high sensitivity of our method. Results disaggregated for the six pathways related to FTD are shown in Additional file 1.

Table 4 presents also the relative ranking of target pathway reported by the different methods. Gene sets having the same *p* value receive the same rank. The gene sets with the lowest *p* value are ranked first, and so forth. Relative rankings are computed by dividing the absolute rank by the number of unique *p* value categories and multiplied by 100 (i.e., percentile rank). Among all methods, SEMgsa() perform the best with a 10th position for the COVID-19 pathway and a median ranking of 39 for FTD. Same ranking for FTD is reported by DEGraph but with a *p* value larger than threshold (0.05). Similar performance is reported by topologyGSA for Coronavirus data, with a ranking of 13 but with poor ranking for FTD (58). Despite the good sensitivity, NETgsa shows poor prioritization results.

ORA method has poorer performance in terms of both sensitivity and prioritization because this type of approach only works when the magnitude of mean changes between conditions is large.

**Table 4:** Benchmark results on Coronavirus disease (COVID-19) and frontotemporal dementia (FTD)

Metrics	Method	Disease	
		Coronavirus disease (COVID-19)	Frontotemporal dementia (FTD)*
Sensitivity	SEMgsa()	$< {\textbf {0.001}}$	$< {\textbf {0.001}}$
	DEGraph	0.771	0.653
	NetGSA	0.011	0.563
	ORA	0.709	0.375
	PathwayExpress	0.444	0.981
	TopologyGSA	$< 0.001$	0.740
Prioritization	SEMgsa()	**10**	**39**
	DEGraph	90	39
	NetGSA	63	44
	ORA	83	56
	PathwayExpress	32	100
	TopologyGSA	13	58

*Since the term Frontotemporal lobar degeneration (an alias for FTD; KEGG ID: H00078) is associated to 6 KEGG pathways, sensitivity and prioritisation metrics have been aggregated by taking the median. Results for SEMgsa() have been highlighted in bold

### Simulation results

**Fig. 2:**
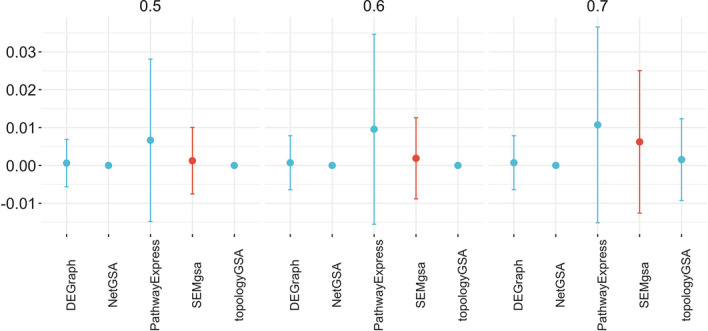
Average type I error on the 10 KEGG pathways grouped by method and mean signal on simulated data. Average type I error together with standard deviation across simulations is displayed for each method. Lower type I error indicates better performance. At the 0.05 significance level, all methods control the type I error rate across the 10 pathways under different level of mean signal

**Fig. 3:**
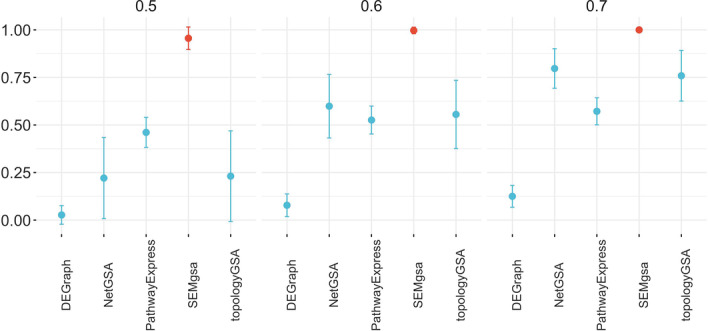
Average statistical power on the 10 KEGG pathways grouped by method and mean signal on simulated data. Average power together with standard deviation across simulations is displayed for each method. Higher power indicates better performance. SEMgsa stands out among all with 90–100% power across simulation. NetGSA and topologyGSA get close to SEMgsa with about 75% statistical power only with differential mean level of 0.7

We summarize the relative performance of all methods based on false positive rate and power results on the subset of pathways associated to the target disease (see “Data simulations” section). We focused on those metrics under the betweenness, community and neighborhood dysregulation design. Metrics were evaluated on 100 simulation replications and were summarized grouping results by mean signal and dysregulation design with the aim of grasping differences in the behavior of GSA methods under different experimental conditions. Error plots (see Figs. 2, 3) show the mean of those aforementioned metrics grouped by either mean signal or topology design together with their standard deviations across simulations. SEMgsa() is highlighted as red, compared to the others colored blue. Comparison figures under different mean signal are reported within the main discussion. More comparisons under different topology designs are available in Additional file 1.

Figure 2 shows that, at the 0.05 significance level, all methods control the type I error rate across the $q_{0}$ pathways, selected among the $K - q_{1}$ pathways with $\le 1$ dysregulated genes under different level of mean signal. This procedure results for betweenness topology design in $q_{0} = 16$, for community in $10 \le q_{0} \le 30$ (note that we have a range of numbers given the random sampling of genes from the community with the highest proportion of members) and neighbourhood in $q_{0} = 14$. Type I error is defined as the average proportion of simulations where the method falsely rejects the null hypothesis of no enrichment. It’s worth noticing that PathwayExpress’ type I error rates show a wider distribution, followed by SEMgsa() which enlarges its error bands in correspondence of the highest mean signal. However, the error rates of the latter methods are near 0 and below the nominal threshold of 0.05. All other techniques appear to have conservative type I error rates. Type I error results have been further investigated for SEMgsa(), showing the ability of the proposed method to capture the signal also at lower values (smaller than 0.5). As a result, given the high sensitivity of SEMgsa(), the higher the value of the mean signal, the higher the rate of false positives. Same results seems not to be shared by the other methods, showing the need of higher differential expression (higher than 0.5) to achieve acceptable performance.

Statistical power of different methods has been investigated in terms of average proportion of simulations where the method correctly rejects the null hypothesis of no enrichment. The higher the power, the better. Figure 3 shows that SEMgsa() stands out among all with 90–100% power across simulations. PathwayExpress reaches a position around 50% statistical power for all the level of mean signal. The same could be stated summarizing the results by dysregulation design, with about 75% statistical power only under the betweenness design (see Additional file 1). As stated previously, the higher the mean signal, the higher the ability of the methods to correctly reject the null. NetGSA and topologyGSA get close to SEMgsa() with about 75% statistical power only with differential mean level of 0.7 (Fig. 3). Slightly better results are reported from both methods with respect to the betweenness design (see Additional file 1). DEGraph instead, is placed at the bottom of the graph for most of the comparisons (statistical power near 0).

Among the methods compared, SEMgsa() has the best overall performance.

## Discussion

Topology-based approaches exhibit greater statistical power in finding pathway enrichment, according to earlier studies [39, 40]. However, several limitations may affect user experience in terms of computational efficiency.

SEMgsa() represent a topological based and self-contained hypotesis method, in line with NetGSA, DEGraph and topologyGSA. Three main points make SEMgsa() more valuable for users than existing GSA methods:

- *Exploiting pathway information*: Existing methods have specific input requirements about pathway topology. TopologyGSA, for instance, only works for pathways whose topology is a DAG and whose size is less than the required number of samples in the two conditions/groups. If a pathway has multiple connected components, DEGraph will check to see if the means vary for each connected subgraph. Without taking into account singletons, NetGSA fits a linear mixed model for each condition. Overcoming this limitations, SEMgsa() accepts as input directed and/or undirected networks that define pathway interconnectedness. Inside SEMgsa() workflow, the function SEMrun() maps the expression data onto the input graph corresponding to each pathway and converts it into a SEM. Node-level perturbation is evaluated according to the specified binary group variable (i.e. case/control) by fitting a “common” model to evaluate group effects on graph nodes. In this way, adding group-nodes and group-genes edges (see Fig. 1), the pathway with several components and singleton genes becomes a connected graph, allowing to exploit all available pathway information.
- *Higher sensitivity to pathway perturbation*: Existing methods, like ORA, save as output of GSA a list of DEGs for each pathway, recovered from the input named vector containing log2 fold-changes of the differentially expressed genes. The latter is obtained from the differential expression analysis done on the gene expression data, where the genes with an adjusted (for multiple comparisons) *p* value smaller than a pre-specified cutoff are considered as DEGs. The adjustment process is highly dependent on the number of tests performed, for example Bonferroni adjusted *p* values are calculated by multiplying the original *p* values by the number of tests performed. SEMgsa() fits a SEM model for each tested pathway. For source and singleton genes, marginal tests of traditional DEGs analysis are applied while for connections and sink genes conditional testing, given the parents are used. When there is a direct group effect, conditioning increases power and reduces gene variability. As a result, the topological structure of the pathway improves the precision of the inference. Furthermore, the significance of node-level perturbation is computed within each pathway and the adjustment procedure for *p* values is less stringent given the smaller number of tests. This choice allows to obtain higher sensitivity to pathway perturbation. Thus, from the output of SEMgsa() we can extract a seed list of DEGS for each pathway that can be useful to discover novel disease-associated interactions. A further step could be to extract a Steiner tree, mapping the DEGs on the union of the KEGG pathways and finding a connected subgraph such that the additional nodes (Steiner or connector nodes) connecting seed nodes (terminal nodes) minimize the sum of the weight of every edge in the subgraph (i.e., maximizing edge perturbation between disease nodes). Then, fitting the filtered active (perturbated disease) module with SEMrun(), we can obtain a perturbed backbone regarding the disease of interest, where important connectors or clusters of genes can be identified [16, 17].
- *Index for overall pathway perturbation*: Among the existing methods, only SPIA reports the direction in which the pathway is perturbed (activated or inhibited), exploiting a posteriori pathway information obtained from hypothesis testing. Like SPIA, SEMgsa() outputs a column summarising overall pathway perturbation, but combining also a priori information obtained from biological databases (up- or down-regulation of genes derived from KEGG) to a posteriori information obtained from the analysis of gene expression data (node perturbation obtained from SEMgsa()). The combination between these flow of information allows to better define the direction of gene perturbation. Table 5 provides the results for perturbation index with respect to benchmark data. COVID-19 pathway is associated to an increase in activation in cases with respect to control group (“up act”); same result can be stated for two out of six pathways (Neurotrophin signaling pathway and MAPK signaling pathway) regarding Frontotemporal dementia. Notch signaling pathway is associated to a decrease in aactivation in cases with respect to control group (“down act”). Note that the NA are reported for the networks without + 1 or $-1$ edge weights in the adjacency matrix, resulting into no calculation for the combinatorial measure.

**Table 5:** Overall pathway perturbation of KEGG pathways related to Coronavirus disease (COVID-19) and frontotemporal dementia (FTD)

Disease	KEGG pathway	Pert
Coronavirus disease—COVID-19	Coronavirus disease—COVID-19	Up act
Frontotemporal dementia (FTD)	Protein processing in endoplasmic reticulum	Up act
	Endocytosis	NA
	Neurotrophin signaling pathway	Up act
	Wnt signaling pathway	NA
	MAPK signaling pathway	Up act
	Notch signaling pathway	Down act

## Conclusions

We have shown that SEMgsa() is easily accessible to common users and provides robust results under several experimental conditions. It obtains external pathway information solving the problem common to many topology-based methods but offering better statistical power and prioritization results, while also controlling for type I error.

We believe that SEMgsa() can be a valuable tool for practitioners, also when undertaking complex pathway enrichment analysis.

## Availability and requirements

Project name: SEMgsa()(**SEMgraph** package)

Project home page: https://github.com/fernandoPalluzzi/SEMgraph

Operating system(s): Platform independent

Programming language: R

License: GNU General Public License version 3 or higher (GPL $\ge$ 3)

Restrictions for non-academic use: None

## Supplementary Information

**Additional file 1.** The file contains additional figures and tables related to the main text. **Fig. S1**. Average type I error on the 10 KEGG pathways grouped by method and topology dysregulation design on simulated data. **Fig. S2**. Average statistical power on the 10 KEGG pathways grouped by method and topology dysregulation design on simulated data. **Table S1**. Disaggregated results for Coronavirus disease (COVID-19) and frontotemporal dementia (FTD).

## Data Availability

Source code is available in https://github.com/fernandoPalluzzi/SEMgraph/tree/master/SEMgsa_replication. The datasets analysed during the current study are available in that repository under the Data/ directory (https://github.com/fernandoPalluzzi/SEMgraph/tree/master/SEMgsa_replication/Data/).

## Abbreviations

BH: Benjamini–Hochberg
CAMERA: Correlation adjusted MEan RAnk gene set test
DE: Differentially expressed
DEG: Differential expression gene
DNAme: DNA methylation
DRN: Differential regulated node
FCS: Functional class scoring
FDR: False discovery rate
FTD: Frontotemporal dementia
GSA: Gene set analysis
GSEA: Gene set enrichment analysis
NetGSA: Network-based gene set analysis
ORA: Over-representation analysis
RICF: Residual iterative conditional fitting
SE: Standard error
SEM: Structural equation models
SPIA: Signaling pathway impact analysis
TB: Topology-based
