# Supplementary material for: SEMgsa: topology-based pathway enrichment analysis with structural equation models
Source: BMC Bioinformatics. 2022 Aug 17;23:344. doi: 10.1186/s12859-022-04884-8 (PMC9385099; doi:10.1186/s12859-022-04884-8)
Supplement: Supplementary file 1 — Additional file 1. The file contains additional figures and tables related to the main text. Fig. S1. Average type I error on the 10 KEGG pathways grouped by method and topology dysregulation design on simulated data. Fig. S2. Average statistical power on the 10 KEGG pathways grouped by method and topology dysregulation design on simulated data. Table S1. Disaggregated results for Coronavirus disease (COVID-19) and frontotemporal dementia (FTD). [file 12859_2022_4884_MOESM1_ESM.pdf]

Additional file 1 for "**SEMgsa**: topology-based pathway enrichment analysis with Structural Equation Models" by Grassi M and Tarantino B

This supplementary document reports additional figures and tables on relative performance of GSA methods. Error plot shows the mean of type I error (Figure 1) and power metrics (Figure 2) grouped by topology design. SEMgsa is highlighted as red, compared to the others colored blue. Table 1 shows disaggregated sensitivity and prioritization results for benchmark analysis.

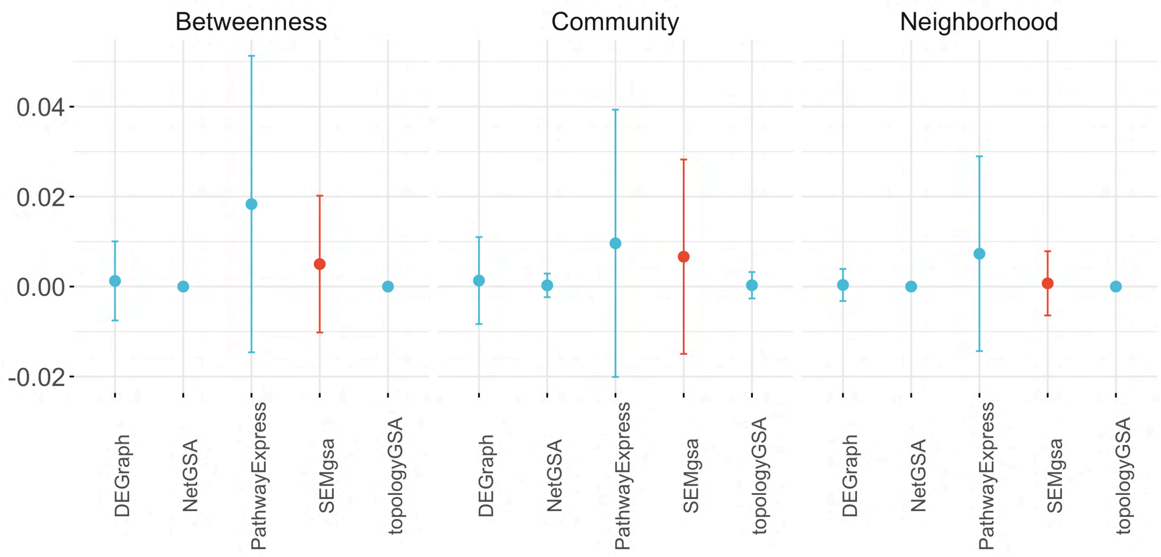

Figure 1: **Average type I error on the 10 KEGG pathways grouped by method and topology dysregulation design on simulated data.** Average type I error together with standard deviation across simulations is displayed for each method. Lower type I error indicates better performance. At the 0.05 significance level, all methods control the type I error rate across the 10 pathways under different topology dysregulation designs.

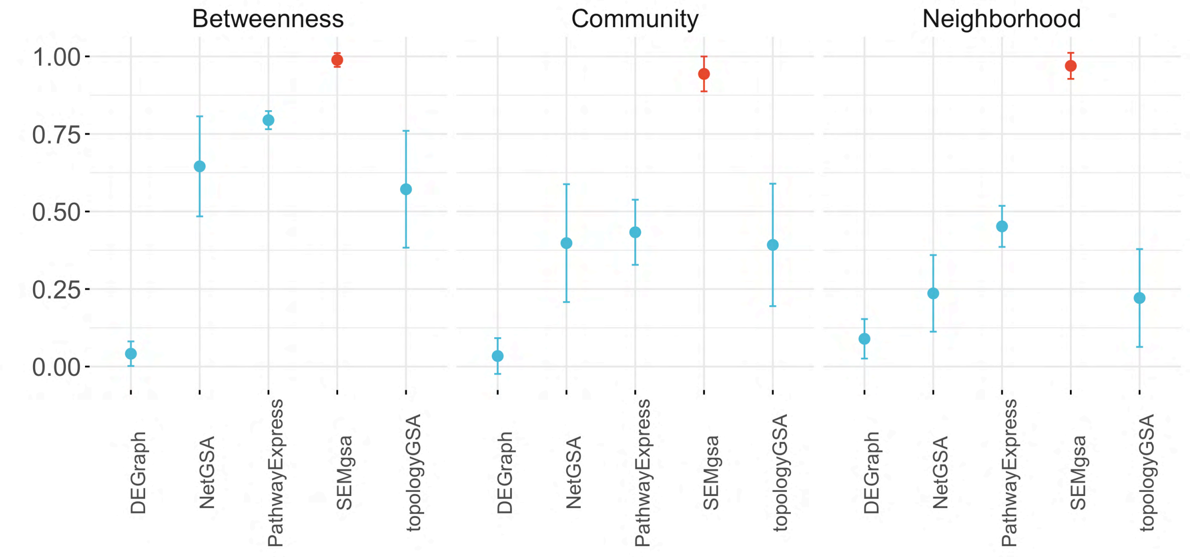

Figure 2: **Average statistical power on the 10 KEGG pathways grouped by method and topology dysregulation design on simulated data.** Average power together with standard deviation across simulations is displayed for each method. Higher power indicates better performance. SEMgsa stands out among all with 90%-100% power across simulation. PathwayExpress, NetGSA and topologyGSA perform slightly better under the betweenneess dysregulation design, reaching about 60%-70% statistical power.

Table 1: Disaggregated results for Coronavirus disease (COVID-19) and Frontotemporal Dementia (FTD).

Disease	KEGG pathway	Method	Sensitivity	Prioritization
COVID-19	Coronavirus disease - COVID-19	DEGraph	0.771	90
	Coronavirus disease - COVID-19	NetGSA	0.011	63
	Coronavirus disease - COVID-19	ORA	0.709	83
	Coronavirus disease - COVID-19	PathwayExpress	0.444	32
	Coronavirus disease - COVID-19	SEMgsa	0	10
	Coronavirus disease - COVID-19	topologyGSA	0	13
FTD	MAPK signaling pathway	DEGraph	0.556	31
	MAPK signaling pathway	NetGSA	0.703	67
	MAPK signaling pathway	ORA	0.323	45
	MAPK signaling pathway	PathwayExpress	0.981	100
	MAPK signaling pathway	SEMgsa	0.007	53
	MAPK signaling pathway	topologyGSA	NA	NA
	Protein processing in endoplasmic reticulum	DEGraph	0.751	46
	Protein processing in endoplasmic reticulum	NetGSA	0.331	1
	Protein processing in endoplasmic reticulum	ORA	0.403	62
	Protein processing in endoplasmic reticulum	PathwayExpress	0.981	100
	Protein processing in endoplasmic reticulum	SEMgsa	0	5
	Protein processing in endoplasmic reticulum	topologyGSA	0.623	33
	Endocytosis	DEGraph	0.827	62
	Endocytosis	NetGSA	0.826	87
	Endocytosis	ORA	0.175	15
	Endocytosis	PathwayExpress	0.981	100
	Endocytosis	SEMgsa	0	8
	Endocytosis	topologyGSA	NA	NA
	Wnt signaling pathway	DEGraph	0.279	8
	Wnt signaling pathway	NetGSA	0.394	14
	Wnt signaling pathway	ORA	0.445	70
	Wnt signaling pathway	PathwayExpress	0.981	100
	Wnt signaling pathway	SEMgsa	0.002	43
	Wnt signaling pathway	topologyGSA	NA	NA
	Notch signaling pathway	DEGraph	0.435	20
	Notch signaling pathway	NetGSA	0.755	82
	Notch signaling pathway	ORA	0.403	62
	Notch signaling pathway	PathwayExpress	0.981	100
	Notch signaling pathway	SEMgsa	0.081	77
	Notch signaling pathway	topologyGSA	NA	NA
	Neurotrophin signaling pathway	DEGraph	0.823	61
	Neurotrophin signaling pathway	NetGSA	0.423	20
	Neurotrophin signaling pathway	ORA	0.346	50
	Neurotrophin signaling pathway	PathwayExpress	0.981	100
	Neurotrophin signaling pathway	SEMgsa	0.002	40
	Neurotrophin signaling pathway	topologyGSA	0.856	82
